# What Kills the Hindgut Flagellates of Lower Termites during the Host Molting Cycle?

**DOI:** 10.3390/microorganisms5040082

**Published:** 2017-12-18

**Authors:** Christine A. Nalepa

**Affiliations:** Department of Entomology, North Carolina State University, Raleigh, NC 27695-7613, USA; christine_nalepa@ncsu.edu; Tel.: +1-919-233-8214

**Keywords:** symbiosis, microbiome, caste determination, polyphenism, molt, eusociality, interdependency, cyst, hormones

## Abstract

Subsocial wood feeding cockroaches in the genus *Cryptocercus*, the sister group of termites, retain their symbiotic gut flagellates during the host molting cycle, but in lower termites, closely related flagellates die prior to host ecdysis. Although the prevalent view is that termite flagellates die because of conditions of starvation and desiccation in the gut during the host molting cycle, the work of L.R. Cleveland in the 1930s through the 1960s provides a strong alternate hypothesis: it was the changed hormonal environment associated with the origin of eusociality and its concomitant shift in termite developmental ontogeny that instigates the death of the flagellates in termites. Although the research on termite gut microbial communities has exploded since the advent of modern molecular techniques, the role of the host hormonal environment on the life cycle of its gut flagellates has been neglected. Here Cleveland’s studies are revisited to provide a basis for re-examination of the problem, and the results framed in the context of two alternate hypotheses: the flagellate symbionts are victims of the change in host social status, or the flagellates have become incorporated into the life cycle of the eusocial termite colony. Recent work on parasitic protists suggests clear paths for exploring these hypotheses and for resolving long standing issues regarding sexual-encystment cycles in flagellates of the *Cryptocercus*-termite lineage using molecular methodologies, bringing the problem into the modern era.

***‘One in general does not kill the goose that laid the golden egg’***

[1]

## 1. Introduction

Overwhelming evidence is now available from morphological characters and genomic analyses of both hosts and symbionts that termites are a clade nested within the cockroaches, with the wood-feeding, subsocial cockroach *Cryptocercus* as their undisputed sister group [2,3,4,5]. A prominent feature uniting *Cryptocercus* with the termites is a symbiotic relationship with single-celled anaerobic eukaryotes living in the enlarged hindgut paunch of the insects. These gut protists fall into two main groups traditionally known as flagellates—the Phylum Parabasalia and the Order Oxymonadida (Phylum Preaxostyla) [6], with the former historically divided into the trichomonads and hypermastigotes. Nearly 450 distinct flagellate species have been described in the termite and *Cryptocercus* hosts that have been investigated (see [7,8] for a catalogue of those reported). The morphological characters historically used to classify species, however, are proving in many cases to be inadequate, and new species and relationships are continually being described based on molecular sequencing techniques, e.g., [9,10,11,12,13].

The flagellates of the two host insect groups have similar taxonomic affiliations, and their distributions are either limited, or they cannot be found in other hosts or in the free-living state [2,8,14]. It has been long thought [15,16] and is now strongly established that divergence of the flagellates took place in a common ancestor of *Cryptocercus* and termites, currently estimated as occurring ~170 mya [17]. Intergenerational transmission is vertical [18,19]: they are transferred from parents to offspring via proctodeal (anal) trophallaxis (the hatchlings feed on parental hindgut fluids) in both *Cryptocercus* and termites; in the latter during the incipient, subsocial stage. In both taxa, the parents are the source of all flagellates for a given social group. In termites, however, the flagellates are re-circulated among colony members via trophallaxis once the colony becomes established. As expected in vertically transmitted symbionts, flagellate communities of different host lineages indicate co-speciation, with possible horizontal shifts due to stochastic, dietary or ecological effects [20,21,22,23].

Functionally, the gut microbiota partner with the host and each other to interactively degrade plant polymers, fix atmospheric nitrogen, synthesize amino acids, and recycle nitrogenous waste products [24,25,26]. Despite the dominance of the single celled eukaryotes, the *Cryptocercus*/termite hindgut system is clearly a multiple symbiosis that involves flagellates, bacteria, and archaea [27]; all organisms involved are symbionts as well as mutualists [8]. The vast majority of prokaryotes in the gut (up to 85%) are associated with the protists [28,29] and exhibit a high level of integration with their flagellate hosts: bacteria or spirochaetes are attached to the cell surface, or located in the cytoplasm or nucleoplasm. Further prokaryotes live free in the gut fluid or are attached to the gut wall of the insect host [27,30,31,32,33,34,35,36,37,38,39]. Clearly the *Cryptocercus*/lower termite gut microbiome is an interdependent, multifactorial, synergistic system whose subtle dynamics cannot be analyzed as simple binary interactions. That being said, the focus here is on the flagellates, which not only drive cellulose degradation [40,41], but also are more exacting in their requirements than prokaryotes [42].

Recently, significant advances have been made in the understanding of these host-symbiont relationships, prompted by the application of new, sophisticated technologies that are independent of microbial culturing. Well over 3000 primary articles and 150 reviews that address termite symbioses were published in the period 2000–2014, inclusive [8]. Emphasis has been primarily on identification of the components of the microbiota in a phylogenetic context, and of individual biochemical functionality and interdependence. Our increased understanding of the sophistication of the microbiome, however, is not matched by an understanding of how it is integrated into the biology, behavior and life history of the insect host.

The environment in which the flagellates live is unusually homeostatic and closely comparable from host to host [43]. The protists are housed in the gut of an individual insect, living within a family (*Cryptocercus*) or colony (termites), which is lodged within the buffered environment of a nest. They are supplied with a steady stream of food, in a liquid, temperature-controlled, safe haven. Their world, then, is fairly constant, with one notable exception: the molting cycle of their insect host.

If the relationship of these two insect taxa to their gut fauna during developmental ontogeny is compared, it is obvious that there has been an evolutionary change during the host molting cycle. In both *Cryptocercus* and lower termites, neonates acquire the flagellates from a parent or a sibling (the latter in established colonies of termites); the symbiosis is established at about the third instar in both taxa. The big difference lies in what subsequently occurs during the molting cycles of the developing juvenile host. After the symbiosis is established in *Cryptocercus*, it is retained through all subsequent ecdyses; it is never lost under natural conditions [15,44,45]. Developing nymphs of this cockroach are thereafter nutritionally autonomous and capable of a solitary lifestyle. In termites, however, the large flagellates die prior to subsequent ecdyses and must be re-acquired by feeding on the hindgut fluids of a nest mate. Unlike most vertically transmitted symbioses [46] then, developing termites have an aposymbiotic phase with respect to the larger gut flagellates. If cockroaches in the genus *Cryptocercus* are used as a model of the termite ancestral state, that shift in the host-symbiont relationship can be pinned to a specific node of the Dictyopteran phylogenetic tree. This paper is an attempt to explore the foundations of that evolutionary transition.

## 2. Why Is Death of Protists in Termites of Significance?

Among eusocial insects, the symbiosis with these gut flagellates is a feature unique to the *Cryptocercus*-termite lineage. Historically, the death of these flagellates at molt was thought to be a key factor associated with termite eusocial origins, e.g., [47,48] but more recent work habitually excludes its influence on social structure. Any hypothesis that ignores this termite specific life history characteristic, however, is unsatisfactory and serves to emphasize the current deep-seated bias in analyzing Isopteran eusocial origins in terms of hymenopteran attributes [49,50]. A re-examination of this symbiotic relationship during host molt is overdue for two compelling reasons: first, because the need to re-acquire the symbiosis after molt precludes independent living by termite individuals, and second, because current literature rarely describes the symbiotic relationship during the host molting cycle in either insect taxon accurately.

### 2.1. Interdependence

In both *Cryptocercus* and lower termites there are two levels of dependence. First, individuals rely on gut flagellates to metabolize and supplement their wood diet; and second, neonates in both taxa rely on their parents (in incipient colonies of termites) for vertical transfer of the symbionts. In termites alone, there is a third level of dependence: because the flagellates die at molt, developing termite individuals do not survive unless they have access to the hindgut fluids of a relative [51]. The dependence on gut flagellates to help metabolize their wood diet, combined with the periodic death of these symbionts precludes independent living in termites. If the host-symbiont relationship in *Cryptocercus* is used as a model of the termite ancestral state, previously independent insects evolved interdependency. Up to the threshold when the protistans began dying during the termite molting cycle, eusociality in termites may have been reversible, but in interdependent organisms the fitness of one depends on the fitness of another, making cooperation the best strategy [52]. The death of protists at molt was a ‘tipping point’ [51], in that beyond that threshold, intrinsic processes in the system drove accelerating change [53].

### 2.2. Misconceptions

**Figure 1:**
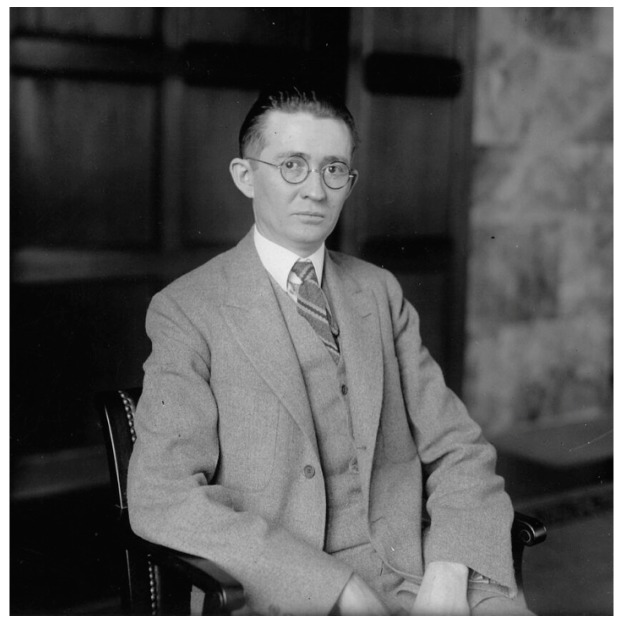
Lemuel Roscoe Cleveland (1892–1969). Smithsonian Institution Archives, Image #SIA2008-0153. The Smithsonian has granted permission for use.

How the gut symbiosis is established in neonates of *Cryptocercus* and lower termites, and how the symbiotic bond between the host and its gut flagellates is either maintained, or broken and subsequently re-established during the host molting period are consistently misunderstood in current literature [51]. Prior to the 1980s, the confusion was understandable because Cleveland et al. (Figure 1) [15], the early authorities on these flagellates, did not understand the social structure and behavior of *Cryptocercus*; rather, these authors assumed two things about the natural history of the cockroach based on their observations of gut flagellates during *Cryptocercus* molting cycles. The first assumption was that neonates of *Cryptocercus* acquired their gut symbionts by feeding on the fecal pellets of recently molted older siblings, because these pellets contained resistant stages of the gut protists. The second assumption was that, because adults do not molt (and therefore their feces would not contain resistant stages of the symbionts), an adult pair of these cockroaches could not found a colony independently. These authors speculated that new colonies were formed by a ‘combined creeping migration’ of adults and older nymphs to a new log. Cleveland et al. ([15] p. 209) did not rule out that trophallaxis may play a role in establishing the gut microbiota in neonate *Cryptocercus*, but they did not observe the behavior during their studies.

Nutting [44] was the first to question the described mode of symbiont transfer in *Cryptocercus*. A subsequent study was done on adults collected in the field as mated pairs [54], suggesting that adults may be able to found colonies independently. In 1983, field work by Seelinger and Seelinger [55] demonstrated that molting older siblings were not present in the nest when *Cryptocercus* egg cases hatch; the typical family consists of a pair of adults with a single cohort of offspring. This social structure, together with the observation of parent to offspring vertical transmission of gut symbionts via proctodeal trophallaxis, was subsequently supported by Nalepa [56] and Park et al. [57]. The subsocial, semelparous life history of *Cryptocercus*, including parent to offspring vertical transmission of gut symbionts, is now well established, and it is generally accepted that extended parental care in an ancestral cockroach modeled after *Cryptocercus* was the key basal condition in the pre-eusocial termite ancestor [8]. Misconceptions regarding the host-symbiont relationship in this lineage nevertheless continue to cloud interpretations of its evolutionary trajectory, e.g., [58,59,60].

## 3. Molting in *Cryptocercus*

The gut microbiota of insects have a unique challenge not faced by those in vertebrate taxa. Because the insect hindgut lining is ectoderm, it is shed along with the rest of the exoskeleton during the molting period throughout host development. Consequently, insect gut microbiota may require measures to cope with the conditions of the digestive tract during this time. As *Cryptocercus* enters its molting period, rising titers of the molting hormone ecdysone trigger the sexual/encystment (SE) cycles (addressed below) of the flagellates in the gut. During the SE cycle, the protistans become more resistant by producing cysts, pseudocysts, or by employing other mechanisms. The lining of the cockroach hindgut is then shed whole, but retained within the host body. The resistant flagellates are then held within the old gut lining (intima) until formation of the new lining is completed. The old intima ruptures, and the protists escape into and repopulate the gut lumen. The old hindgut lining subsequently breaks into small pieces, and is voided in fecal pellets along with some resistant protists [15,61]. Although these protists may pass with host feces shortly before or after molting (in some hosts half or more, in others just a few), they play no role in intergenerational transmission under natural conditions.

Prior to the work on social structure of *Cryptocercus* in the 1980s, it was thought that SE cycles in flagellates of *Cryptocercus* were rooted in a fecal-oral mechanism of transmission between generations. The cysts were considered mandatory to withstand the stress of being subjected to the environment outside the insect host, i.e., in fecal pellets lying on the floor of the nest. We now know that flagellates are transferred to neonates via ingestion of the trophozoite stage in the hindgut fluids of parents. After their hindgut microbiota have become established at about the third instar, trophallactic behavior in young nymphs of *Cryptocercus* ceases. The fact that some cysts may be found in the fecal pellets of post-molt juveniles of *Cryptocercus,* however, suggests that contemporaneous members of the family may ingest some of these; trophozoites that subsequently emerge from these cysts are probably re-integrated into the existing gut microbiota of the coprophage. Feces are a part of the diet in most cockroaches, including *Cryptocercus* [18,62]. However, because all flagellates in a given family originate with the parents, no new flagellate taxa would enter the social group via coprophagy.

## 4. Molting in Termites

The most common error regarding the disappearance of the protistans in molting termites is that the hindgut lining is excreted whole with the protists still in it; prevalent fallacies regarding how the symbiosis is re-established after molt is that it is restored by coprophagy or by feeding on an intact, shed hindgut lining. With a few possible exceptions (discussed below), however, the flagellates are dead and gone from the hindgut prior to the molt of their termite host [44,63,64,65,66,67]. The symbionts are not ‘shed’, ‘discarded’ or ‘cast’ with the hindgut lining. The gut is devoid of flagellates seven days before molt in *Kalotermes flavicollis* [68] and six days before molt in *Coptotermes formosanus* [69]. The physical process of molt in termites is the same as in *Cryptocercus*. The lining of the hindgut is shed but retained within the body for two or three days; it later breaks up and is voided. Consequently, the gut protists cannot be re-acquired by a post-molt termite via feeding on its own shed exuvium, that of a nestmate, or by feeding on feces. The hindgut lining is not attached to the remainder of the shed exuvia [70], and termite feces never contain active flagellates, cysts, or other resistant stages [15,43,47,61,64,71]. The sole mechanism of refaunation in a newly molted termite is via interaction with a conspecific: repeated proctodeal trophallaxis with intermolt donor nestmates [51]. If *Cryptocercus* is used as a model of the termite ancestor, the host-symbiont relationship during molt changed from retention by the individual host via the behavioral and physiological responses of the flagellates (SE cycles), to retention by the colony via host behavioral interaction with other colony members [18].

One explanation offered for the death of termite flagellates during host molt was that because termites exhibit colony-wide proctodeal trophallaxis, the SE cycle in their flagellates was superfluous and expendable, as the flagellates were never exposed to the outside environment [16]; this explanation is still popular [72,73]. It is the premise of this paper that the directionality of cause-and-effect of this common assumption should be reversed. Instead of colony-wide trophallaxis leading to the death of flagellates at host molt, the early stages of eusociality led to dysfunctional SE cycles in the flagellates and required a behavioral solution on the part of the host: colony-wide trophallaxis [18,51].

## 5. Why Is the Partnership Dissolved at Every Molt in Termites?

It is commonly assumed that abiotic factors in the gut environment are responsible for killing termite protistans during molt, and that encystment by the flagellates in *Cryptocercus* protect them during this period. The flagellates are known to be extremely sensitive to changing conditions of temperature, food, and water [74]; indeed, subjecting *Cryptocercus* and termites to heat, starvation or oxygen is a standard technique for rendering them aposymbiotic for experimental purposes, e.g., [75,76]. The assumption that such conditions kill termite flagellates during molt is therefore understandable, since these symbionts pass through a gauntlet of harsh conditions each time the insect undergoes a molting cycle. The host ceases to feed, and the gut fluid increases in viscosity to a heavy paste [61,77]. Large bubbles also appear in the hindgut ([15] Plate 7), suggesting potential vulnerability to oxygen poisoning. In free living and parasitic protists, stimuli such as these are often the first triggers of a cell differentiation process that leads to encystment, thus enabling resistance to a deteriorating environment [78,79,80]. Starvation, desiccation or oxygen, however, play no role in initiating the SE cycles of the flagellates in *Cryptocercus* [44,81]. The cycle is triggered by rising titers of host ecdysone long before there is a change in the feeding habits of the host or a change in viscosity of the gut fluid [81,82]. Furthermore, SE cycles can be induced with ecdysone in the flagellates of intermolt *Cryptocercus* nymphs as well as in adults, both of which have normal, wood-and fluid-filled guts [45]. The relevant question, then, is why does a rising titer of the molting hormone ecdysone trigger a differentiation process in the flagellates of *Cryptocercus*, but lead to disintegration of the flagellates in termites? To address this question, the host-symbiont relationship at molt displayed by *Cryptocercus* will be examined in detail.

## 6. Flagellate Differentiation during the *Cryptocercus* Molting Cycle

Protists in *Cryptocercus* exhibit two morphologically distinct stages in the life cycle. Intermolt *Cryptocercus* typically harbor the trophozoite stage, which reproduces by mitosis. Flagellates change from asexual to sexual methods of reproduction (i.e., initiate an SE cycle) at the time their host enters its molting period. The molting period of *Cryptocercus* begins with the first appearance of ecdysone in the insect and ends with the disappearance of this hormone. Ecdysone can begin rising as much as 50 days prior to ecdysis, and persists until three days after [81,83,84]. Ecdysis is defined as the behavioral act of shedding the exoskeleton. Note that for simplicity, I am here calling the differentiation events in flagellates during host molt a sexual-encystment (SE) cycle, regardless of the genetic consequences of the cycle and the mechanism of resistance. There is extraordinary variation in the sexual cycle of the flagellates during the *Cryptocercus* molting cycle; likewise, the morphological indications of flagellate resistance vary, both in degree and in the timing of appearance.

### 6.1. Cryptocercus: Variation in Flagellate Sexual Cycles

Cleveland [61] thought that the flagellates in *Cryptocercus* exhibited a variety of sexual forms as extensive as all other protistan groups known at his time. In general, his accounts of the SE cycles in the flagellates indicate little opportunity for genetic recombination. There are reports of remarkable abbreviations of, and variations on, standard sexual processes that are difficult to interpret (summarized in [85,86,87]). In *Pyrsonympha*, for example, meiosis-like reductional divisions can be considered either degenerated meiosis or as precursors of conventional meiosis [88]. Haploid lineages undergo postzygotic meiosis, some with one, others with two divisions [89]. Diploid flagellates show pre-gametic meiosis requiring two divisions in some species and just one in others. Autogamy occurs in a few species. One-divisional meiosis probably exists only in flagellates from *Cryptocercus* (reviewed by Raikov [90]). Even within a given flagellate species, SE cycles do not always follow a uniform pattern. *Leptospironympha eupora*, for example, may encyst either before or after gametogenesis; the usual pattern being encystment following fertilization [91].

Although meiosis is highly conserved in eukaryotes, deviations from the norm are ubiquitous and the evolutionary significance of these deviations is largely unknown. In many protists meiosis is not tightly associated with reproduction [88,92,93], and may have a functional basis in DNA restoration rather than in recombination, i.e., elimination of deleterious mutations, direct repair of DNA breaks, or removal of oxidative DNA damage [94]. Other apparent advantages include recombination fidelity, ploidy reduction and epigenetic resetting (refs. in [95]).

### 6.2. Cryptocercus: Variation in Flagellate Mechanism of Resistance

The flagellates in *Cryptocercus* exhibit the entire spectrum of the morphological indications of resistance during host molt, ranging from those that produce a thick, double-walled cyst to those that undergo a differentiation cycle during host molt but display no physical evidence of a resistant outer wall. Individuals of more than one species can skip an SE cycle altogether but are nonetheless retained during host molt.

True cysts are defined as having a thick hyaline wall formed from the outer layer of the cytoplasm, with an empty space between the inner margin of the cyst wall and the living cytoplasm [96]. Examples of flagellates that clearly form cysts include *Trichonympha* and *Macrospironympha*; Cleveland [82] considered the latter as the best flagellate for studying encystment. The definition of a pseudocyst is more nebulous. A general definition is that it is a compact, resistant, resting form, where the cell rounds up without forming a cyst wall [97]. Cleveland, however, struggled with defining this mechanism of resistance. In *Barbulanympha*, for example, he described the organism as losing water, taking on a hyaline appearance, discarding its flagella, becoming circular and immobile, and becoming capable of withstanding conditions outside the host body. He considered it a ‘clear cut case of protozoa becoming resistant without formation of an external, protective cyst membrane or wall’ but refrained from naming the condition [15]. He later, however, indicated that *Barbulanympha* forms a pseudocyst [98]. *Rhynchonympha* was described as undergoing ‘partial or pseudoencystation’, but it was also noted as ‘sometimes’ forming a cyst [96,99]. Variation within a genus was noted: *Oxymonas nana* forms a cyst, but *O. dorsalis* forms a pseudocyst [89]. *Leptospironympha* typically forms a cyst, but there is no encystment or pseudoencystment in *L. wachula* [91,96]. As in *L. wachula*, *Eucomonympha, Notila* and *Saccinobaculus* apparently undergo sexual cycles with neither encystment nor pseudoencystment [96,100,101].

Finally, Cleveland described instances of flagellates that do not undergo an SE cycle but are nonetheless retained by *Cryptocercus* during molt. Although in most flagellates all individuals undergo an SE cycle when their host molts [61], a small percentage of those in the genera *Trichonympha*, *Leptospironympha* and possibly others do not. These individuals are actively motile, free of wood particles, and location specific: they are always found in the very anterior part of the hindgut [15,61,91]. The number of these individuals that do not undergo an SE cycle is always sufficient to repopulate the gut after molt. Consequently, Cleveland et al. ([15] p. 262) concluded that the function of flagellate encystment was to produce resistant organisms capable of withstanding conditions external to the host, so that they may be transmitted in feces to neonates. Since we now know that these flagellates are vertically transmitted between generations via proctodeal trophallaxis, another possibility suggests itself: cysts in flagellates of *Cryptocercus* are currently of no functional consequence, but are vestiges from a distant gregarious ancestor when externally excreted cysts functioned in horizontal transmission [85]. Fewer than half of the flagellates studied by Cleveland exhibit the thick outer wall associated with a true cyst ([83] Table 2), and the morphological variation in resistance exhibited by the flagellates in this host may represent stages in dispensing with the costly encystment process. Encystment in the infectious vertebrate parasite *Giardia lamblia*, for example, involves pulsed production, processing and secretion of cyst wall material, and neogenesis of encystment specific vesicles [80,102], indicating substantial metabolic and material investment.

## 7. Link between Social Behavior and Symbiont Transmission 

**Figure 2:**
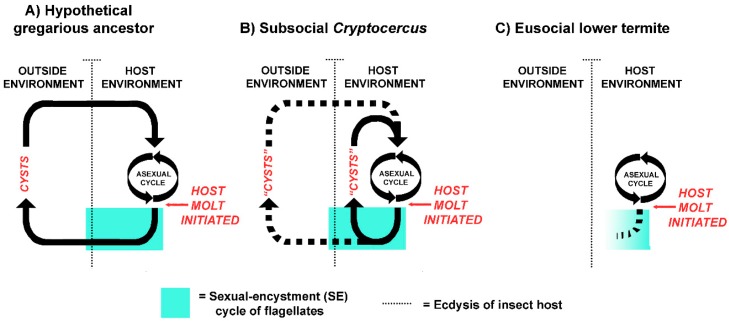
Sexual-encystment cycles of the flagellates in molting individuals of: (**A**) a hypothetical gregarious cockroach ancestor, (**B**) the subsocial cockroach *Cryptocercus*, and (**C**) eusocial lower termites. The symbiont-host relationship logically evolved in sequence, along with shifts in host social behavior, from a distant gregarious cockroach ancestor, to a subsocial *Cryptocercus*-like ancestor, to eusocial termites [18]. In both *Cryptocercus* and termites, the SE cycle has degenerated from the strong encystment pattern likely shown by an ancestor, but in both taxa, protistan numbers and functions are reliably restored after molt, albeit in different ways (retention of protists in individuals of *Cryptocercus*, anal trophallaxis from nestmates in termites). Neither *Cryptocercus* nor termite protists currently have a functional relationship with the external environment. Inspired by Weedall and Hall ([103] Figure 1).

The host-symbiont patterns currently seen in *Cryptocercus* and extant lower termites suggests a logical evolutionary sequence (Figure 2). In a distant gregarious cockroach ancestor, the host-symbiont relationship was not obligate, and the flagellates likely had two distinct differentiated states: the vegetative state inside of the insect host, and the encysted state in fecal pellets in the outside environment. Encystment was a crucial part of the life cycle, as it was the mechanism of horizontal transmission via the fecal-oral route (coprophagy) [18]. As in well-studied parasitic protists from diverse lineages, meiosis and resistance to the outside environment were linked to dispersal from the host [103]; it allowed the flagellates to withstand external conditions if new hosts were not immediately available. Cysts within fecal pellets served as an ‘infection bank’, much like the gregarines that parasitize extant gregarious cockroaches [104,105]. Aggregation behavior of the distant cockroach ancestor served as a mechanism that concentrated both the cysts and the hosts in a limited space.

With *Cryptocercus* as model, proctodeal trophallaxis evolved from this pre-existing intraspecific coprophagous behavior when termite ancestors became semelparous and subsocial, because the physiology of encystment in the flagellates precludes their transfer via cysts in adult feces: adults don’t molt, and older developmental stages are not present in the social group. This was a key point in their evolutionary history, as it assured vertical intergenerational transmission of all pro- and eukaryotic members of the gut microbiome, led to growing interdependence of the host and symbionts, and established the behavioral basis of trophallactic exchanges [18,51]. Parent to offspring proctodeal trophallaxis in both *Cryptocercus* and termites during colony initiation is currently a mechanism of both inoculating neonates with the symbionts and of supplying them with nourishment until their microbiome becomes established. Table 1 summarizes the similarities and differences in the host-symbiont relationship in *Cryptocercus* and lower termites.

## 8. What Changed?

If *Cryptocercus* represents the immediate ancestral condition of termites, how was the transition in the host-symbiont relationship made? Based on his flagellate research, Cleveland’s [61,82] interpretation was that somewhere in the evolution of termites the molting process changed so that the flagellates were killed each time its host molted, instead of switching from asexual to sexual reproduction as they do in *Cryptocercus*. The protistan SE cycle is initiated and synchronized by host ecdysone; this phase of the flagellate life cycle is therefore indirectly dependent on the neurosecretory cells of the host [106]. It is reasonable to assume, then, that a change in host endocrinology at molt had consequences for its symbionts; a change in the developmental trajectory of an insect is invariably rooted in a change in its underlying hormonal physiology.

The origin of termite eusociality is primarily a developmental phenomenon [49,107,108,109]. *Cryptocercus,* like other cockroaches, has a single more or less fixed developmental track: a linear, progressive hemimetabolous development to the adult stage, within a specified, albeit flexible, time frame; size, maturity and chronological age are roughly correlated. Termites deviate from this developmental blueprint in two general ways. First, the vast majority of individuals in lower termite colonies are workers (also called alloparents, helpers), which are functionally sterile, developmentally plastic, immature stages stalled in a small bodied, altricial morphotype ([110] Figure 2); they can molt repeatedly without growth or differentiation [111]. Worker chronological age is disassociated from maturity via progressive, stationary, saltatory, and reversionary molts. As such, these workers exhibit a strong temporal deviation from standard hemimetabolous development at the level of the individual.

Second, workers in lower termites retain the capacity to differentiate into either soldier or reproductive caste phenotypes, in essence shifting from a hierarchical model of ontogeny (egg—embryo–juvenile–adult) to one with alternate pathways. These alternate developmental tracks are rooted in the independent ontogeny of different organs relative to each other [112]. Termite developmental evolution therefore entailed not only a drastic temporal change in the ontogeny of the whole insect, but also changes in the relative developmental timing of different organs within an individual. For example, soldier-specific cuticle formation and the exaggerated growth and cell death associated with forming the morphologically specialized head and mouthparts of this caste occurs during the molting period and inevitably involves ecdysone [113,114,115]. The timing, amplitude and duration of ecdysone pulses are key in initiating and controlling such developmental transitions by activating signaling cascades in target tissues [116]. Most work on the endocrinology of caste determination in termites to date has focused on the role of juvenile hormone (JH), e.g., [111,117,118,119,120,121], but ecdysone and JH act in concert as key endocrine timers in precisely controlling the onset of developmental transitions and in harmonizing gene expression profiles [116,122,123]. The recognition of cross-talk between these two classes of hormones may be key in determining the endocrine basis of termite caste determination [115,124,125] particularly as these hormones are thought to be overlooked players in the epigenetic control of gene expression [126].

In sum, there was extensive remodeling of the nature and timing of endocrine signaling pathways associated with host molt when the subsocial cockroach ancestor became eusocial. Because the ultrastructure, metabolism and gene expression of the flagellates was deeply integrated into the ontogenetic rhythm of their subsocial cockroach host, it is reasonable to assume that, as suggested by Cleveland, a drastic change in host developmental ontogeny had fatal consequences for the flagellates. Support for this suggestion comes from Cleveland’s work on the flagellates over the entirety of his career.

## 9. Titer of Ecdysone and Timing of Events during the SE Cycle

**Figure 3:**
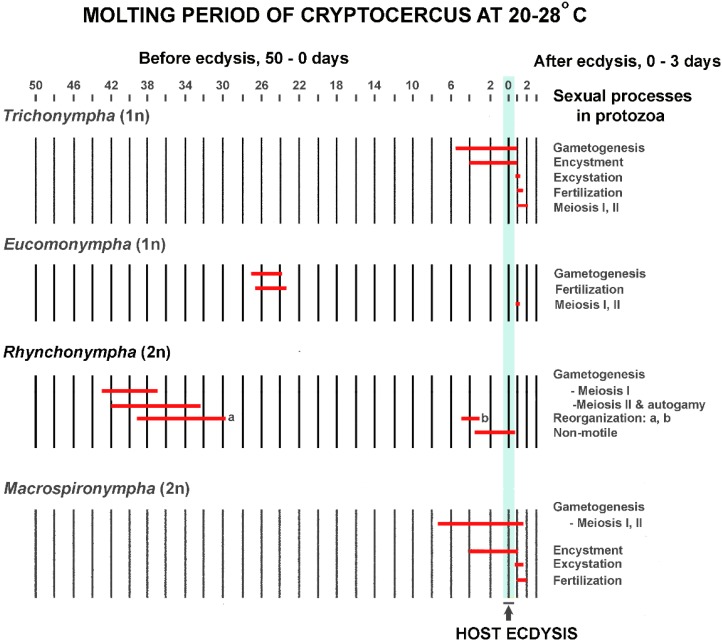
Examples of timing of different phases of the sexual/encystment (SE) cycle in relation to the molting period of the host in four genera of flagellates in the gut of *Cryptocercus punctulatus*. For each genus, the data are summed from several hosts, thus giving the time span during which the processes may occur. In *Rhynchonympha*, ‘reorganization a’ refers to unsynchronized partial re-organization of the centrioles; ‘reorganization b’ refers to synchronized loss and renewal of all the other extranuclear organelles. After Cleveland ([83] Table 2, with permission from John Wiley and Sons).

In *Cryptocercus,* the timing of host hormonal ebb and flow for the flagellate SE cycle is critical to the outcome, just as it is for termite caste determination. From the beginning of the molting period until several hours after ecdysis, the host is constantly changing the hormonal environment in which the hindgut protists live [81]. Each flagellate species has its own time schedule in the host molting process for initiating its SE cycle; this reflects the different thresholds of susceptibility to host ecdysone levels. For example, under natural conditions *Barbulonympha*, *Saccinobaculus* and *Oxymonas* begin gametogenesis 40–45 days before ecdysis of their host, while *Trichonympha* begins this phase of its life cycle 5–6 days before ecdysis [127]. Timing is so precise that the stage of the sexual cycle in well-studied flagellates can be used as an indicator of the host molting cycle [81]; it is rare for a given stage of the cycle in one flagellate genus to overlap in time with that of another [82]. Although the timing and details of the flagellate cycles differ (Figure 3) [83,84], the common thread is that in each studied flagellate they can be initiated with an injection of ecdysone. This occurs if an adult *Cryptocercus* or an intermolt nymph is injected in doses too low to trigger ecdysis in the host [84]. The protists do not have intrinsic factors that initiate the SE cycle [81], and molting cycles of the host and SE cycles in the protistans never occur separately [61].

Ecdysone acts differently on each flagellate genus not only in terms of triggering the SE cycle, but also in orchestrating its details [96]. In some genera of protists, for example, the ecdysone titer must drop before meiosis can begin [66]. Timing of the various events in relation to each other is crucial; they happen in rapid succession, and a slight variation in timing with respect to other events can be lethal [96]. The discarding and renewal of extranuclear organelles during division is one example of the perfectly coordinated and delicately balanced cell mechanics [100,101]. In those flagellates that form a resistant wall or membrane, encystment is physiologically integrated with the sexual cycle. For instance, in *Macrospironympha*, all divisions, haploid gametogenesis as well as meiosis, are synchronized during encystation. If these same divisions occur when the cell is not encysted, synchronization is lacking and the division process speeds up [82].

Transfaunation experiments indicate that once the SE cycle has been initiated by host ecdysone, there is no turning back; the flagellates reach a ‘point of no return’ early in the process. Any hormonal change in titer or timing that interferes with the early stages of the SE cycle blocks or alters all downstream events. If there are more than a few hours difference in the molt cycles of a donor and recipient, a transfer of gut contents results in flagellate degeneration and death [81].

### Period of Vulnerability

Only those flagellates that have initiated the SE cycle are susceptible to hormonal disruption; the beginning and end of the host molting cycle bracket the period of vulnerability [77,81]. Key to the arguments here is that the SE cycle is initiated before there are any visible morphological changes in the flagellates; transfaunation experiments indicate early metabolic modifications that could not be detected using microscopy techniques available at the time [81]. Cleveland and Nutting [81] concluded that the SE cycle includes a 2–3 day ‘conditioning’ period prior to the first visible indications of the cycle when ecdysone is slowly preparing the flagellates for carrying out the numerous unusual and striking differentiations of chromosomes, nuclei, and cytoplasm that subsequently occur [81,96]. If a flagellate is transferred to a recipient host three or more days before the visible SE cycle is scheduled to begin, it will proceed normally; if transferred later it dies [81]. Flagellate death in these cases is not a gradual process like it is when flagellates starve; they were completely broken down in six hours. The protists were observed in various stages of disintegration, beginning with loss of flagellar motility, then progressing with disintegration of the flagella, parabasals, chromosomes and cytoplasm.

As in other one-celled eukaryotes that encyst, morphological differentiation during SE cycles of *Cryptocercus* flagellates is likely coordinated at multiple levels, and involves cascades of up- and down-regulated genes associated with metabolic adjustment, nuclear division, and protein synthesis and transport [102,128,129,130,131]. Combining encystment with co-occurring sexual processes compounds the complexity, as meiosis is controlled by hundreds of genes, genes shared with mitosis as well as those that are specifically meiotic [132]. These processes are likely activated during the early ‘conditioning’ period of the flagellates in *Cryptocercus*, before the SE cycle is visible but when it is already susceptible to host hormonal changes. If so, there is a strong possibility that SE cycles in flagellates may be initiated during juvenile molts of lower termites, but the cycle is aborted with consequent disintegration of the flagellates before there is visible evidence that the cycles were occurring. Cleveland provided evidence that such was the case.

## 10. The ‘Smoking Gun’

Although as a rule, all the large protists die prior to ecdysis in termites, Cleveland documented that some flagellates undergo sexual or sex-like differentiation prior to their death. The phenomenon is especially well studied in the basal termite *Mastotermes darwiniensis*, where the cycles were found in the flagellates *Deltotrichonympha*, *Koruga*, and *Mixotricha* (Table 2). The cycles were rare (1–2% in *Trichonympha*), and always correlated with the termite molting cycle. Although these flagellates were sometimes successful in producing a zygote, it was nonetheless short-lived; the process was always abortive prior to host molt [133]. There is no mention of encystation in Cleveland’s descriptions of these SE cycles, and sexual phenomena could not be documented in the protists of some other termites that were studied during molt, e.g., *Zootermopsis* [44]. Messer and Lee [134], however, fed ecdysone treated paper to workers of *Zootermopsis* and observed both sexual stages and cysts in *Trichonympha*. The evidence suggests, then, that there was not a complete loss of the SE cycle during molt in juvenile stages of termites; there was loss of a functional SE cycle. The underlying machinery is still present and can be activated, even if morphological changes cannot be detected in flagellates examined under a microscope. It is notable that when *Cryptocercus* donor flagellates are transferred to recipient termites, they initiate an SE cycle during termite molt, but then degenerate, just as termite flagellates in the termite host usually do. The fact that these SE cycles abort, with resulting degeneration of the protists parallels precisely the behavior of the sexual protists after they are transferred to another cockroach host either more or less advanced in the molting cycle [44].

The aborted SE cycles of termites during molt are a ‘smoking gun’ that supports Cleveland’s hypothesis: whatever hormonal mechanisms suppress or alter progressive maturational development of the termites also disrupts the SE cycle of termite protists. Flagellate disappearance prior to molt is not a programmed cell death, nor the result of starvation, dehydration or oxygen levels; it stems from an evolutionary change in the host hormonal regulatory network at molt. Recent support for the hypothesis comes from studies of termite physiology. Raina et al. [69] found that, prior to molt, a significant increase in JH titers coincided with the disappearance of the protists in the gut of *Coptotermes formosanus*, while ecdysteroids peaked a day later. Sen et al. [140] showed that juvenile hormone was associated with the down-regulation of protist genes in *Reticulitermes flavipes*, possibly indicating flagellate susceptibility to host hormones.

## 11. Exceptions to the ‘Rule’ in Termites

There are two exceptions to the ‘rule’ of flagellate loss during molt in termites. The first is that in some (but not all) termites, the smaller and simpler protists, specifically those in the genera *Tricercomitus*, *Hexamastix*, and *Streblomastix,* persist through the molting cycle of their host, and undergo unusual morphological modifications during this time [43,64,65,141,142,143]. These genera were not studied in detail by Cleveland, who concentrated his efforts on the large, cellulose-digesting taxa.

More intriguing from an evolutionary standpoint is that there are several reports that the symbiotic association with large, wood-digesting flagellates is often retained by the termite host during its final molt to the alate (adult, imaginal) stage. The imaginal molt differs from those previous in more than one aspect. It is a molt in which the termites acquire adult structures and reproductive competence, thus entailing a different hormonal regulatory network [44]; physiologically, it may be the only ‘normal’, cockroach-like, hemimetabolous molt experienced by termites [49]. This is also when alates prepare for their dispersal flight from the nest. During the imaginal molt much of the gut contents disappears, along with most of the protists; a representative sample of the whole is, however, retained within the sac-like shed intima and later released into the gut lumen of the imago [43,44,71,74,138,142,144]. Voiding of most of the gut contents prior to termite dispersal is thought to be a measure taken to make flight possible [138]. In *Mastotermes darwiniensis*, for example, the hindgut paunch at flight it is just 3% of alate wet weight, while it is typically 22% in two-month-old colonies [145].

Cleveland [133] reported sexual cycles in flagellates during this final molt (*Trichonympha magna* in *Porotermes*), and others report complex morphological transformations that suggest sexual phenomena [65,71,142]. There are a few reports of cysts or pseudocysts formed during the imaginal molt [65,71], but the consensus seems to be that the evidence for these is slim [72,74].

In one studied termite (*Cryptotermes lamanianus*), recently eclosed alates were found to be devoid of flagellates and therefore dependent on proctodeal feeding from nest mates to replenish their symbionts prior to flight from the nest [71]. There are also reports that *Reticulitermes lucifugus* retains its flagellates during the imaginal molt in some geographic locations but not others [65,71]. Currently, it appears that at least some termites retain their flagellate symbionts during their final molting cycle, the flagellates may undergo sexual processes during that period, but they probably do not encyst. How they survive the final molt to the alate stage is unknown, but the answer may lie with the flagellates of *Cryptocercus* that survive host molt, but for which there is no evidence of encystation.

### Two Types of Encystment?

A sidetrack to the main arguments is in order here, because Dolan et al. [146] recently described cysts of the large hypermastigote *Staurojoenia assimilis* in the gut of the drywood termite *Neotermes*. These authors sampled ‘more than three dozen termites’, and found purported cysts (2% of cells) in a single termite of unreported developmental status. The cysts were mixed in among normal swimming cells of the same flagellate species. No cell division was observed, and the photographed cyst did not exhibit a space between the cyst wall and the cytoplasm, typical in encysted gut flagellates of *Cryptocercus* [96] and some other protists [130]. Moreover, it was not determined if the examined termite was in its molting period. These cysts, then, cannot be categorized with the flagellate cysts associated with molt in *Cryptocercus*, where nearly 100% of a given species in the host synchronously undergo an SE cycle during the host molting period [45,61,81]. It is fully possible, however, that the flagellates associated with the *Cryptocercus*-termite clade may have more than one type of resistant stage. A protist species can exhibit a variety of cysts: as a regular part of the life cycle (like the reproductive cysts in flagellates of *Cryptocercus* during molt), and those instigated by a variety of different environmental conditions (protective, or resting cysts) [147,148]. Those reported by Dolan et al. [146] may be an example of the latter. Support for the idea of more than one type of cyst comes from the finding that *Cryptocercus punctulatus* can be collected from frozen logs during winter in their Appalachian mountain environment [149]. During that time, their guts are a creamy white color (Nalepa, pers. obs.), indicating that they are not feeding on solid wood [150]. It would be of interest then, to determine how the gut flagellates of *Cryptocercus* are maintained during winter, as protective cysts are a distinct possibility. Cleveland [89] indicated that he observed *Oxymonas* encysted in the gut of *Cryptocercus* in winter, during the asexual phase of this flagellate’s life cycle.

## 12. Hypothesis I. Protists as Victim

The evidence to date suggests two evolutionary hypotheses regarding the death of termite flagellates during the host molting cycle, providing a basis for further studies. The fact that non-functional sexual cycles are still sometimes found in the flagellates of termite juveniles during molt (Table 2) more than 150 million years after their hosts became eusocial supports the idea that the loss of a functional SE cycle may have been imposed on the flagellates by the host.

In general, the partners in a vertically transmitted, obligate symbiosis are not equal. A loss of autonomy on the part of the smaller partner, in this case the flagellates, gives the host full control of the alliance, limiting the evolutionary pathways available to the symbionts. They can neither enter the system from the outside environment, nor re-evolve to free-living status. Eventually the smaller partner progresses to oblivion or to partial incorporation into the host [46,151,152,153]. Evolutionarily, the symbiosis with flagellates in the termite lineage did progress to oblivion: higher termites harbor only bacteria and archaea as gut symbionts [154,155]. Loss of the intestinal flagellates is the single definitive trait separating the higher termites from the lower termites [8], and may be the fourth, final step in the progression depicted in Figure 2. The pattern currently exhibited by flagellates in lower termites may be viewed as the penultimate step in this loss; they exemplify symbionts that lumber along, incompletely adapted to the present environment [151]. Cleveland et al. [84] argued that in flagellates of *Cryptocercus*, host ecdysone had been ‘captured by the protozoa’ and turned to their own ends. If so, perhaps it backfired on them when the hormonal regulation of host development changed.

## 13. Hypothesis II. Coordination of Life Cycles

**Figure 4:**
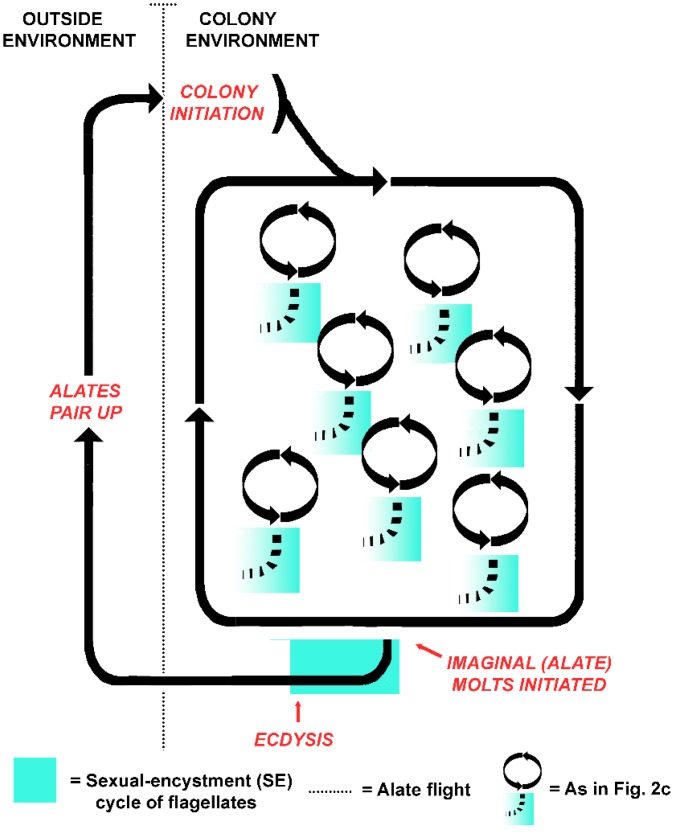
Coordination of the life cycle of a termite colony with the sexual/encystment (SE) cycle of the gut flagellates of individual colony members. During typical molts of colony members, the flagellates exhibit aborted SE cycles and die. During the imaginal molt to the alate stage, the flagellates undergo a functional SE cycle in concert with the reproductive maturation of the termites.

An alternative to the protist as victim hypothesis is that the life cycles of the termite colony and the flagellates became aligned when the host evolved eusociality. Instead of individual host-symbiont fitness, colony-symbiont fitness became coupled, with intermolt termites serving as flagellate reservoirs [51]. An intriguing possibility, then, is that the symbiotic flagellates alternate between sexual and asexual cycles in concert with the colony cycles of their superorganism host (Figure 4). In this model, both the termites and the flagellates are functionally asexual while within the colony, but sexual (in whatever form it takes) when they venture together into the outside environment. Two components of the holobiont became synchronized: the sexual cycles of the gut flagellates, and those of the termite colony, as alates are essentially the gametes of the superorganism. Rather than signaling that the termite flagellates are a victim of the hosts, then, the truncated SE cycles of flagellates in workers in a termite colony exist because the cycles are still functional and of value in termite alates. The crucial time for sexual processes occurs not at individual molt (Figure 2c), but with the founding of new colonies (Figure 4). This accords with the idea that symbiotic associations in social insects are best understood by considering the colony as the host, as the symbionts meet the needs of the colony and not necessarily the individuals that house them [156]. Why would flagellates have retained the mechanism for such an energetically costly process for eons were it merely a dead end?

## 14. Future Prospects

Key to distinguishing between these hypotheses will be to determine the behavior of flagellates during the final, imaginal molt of their termite hosts. Survival and sexual activity of the flagellates should be the primary foci, as encystment may not be a requirement to withstand the host molting period. The behavior of the host at this time is of equal interest. Are alates fed hindgut fluids prior to their dispersal flight to assure an inoculum of symbionts? Or do they abstain because they have retained a representative sample of protists during their final molt, and flight weight is critical? Alates in three termite species studied by Heath [157] are reported to forego any form of nourishment from the time of their last molt until they have paired and begin establishing a new nest.

**Figure 5:**
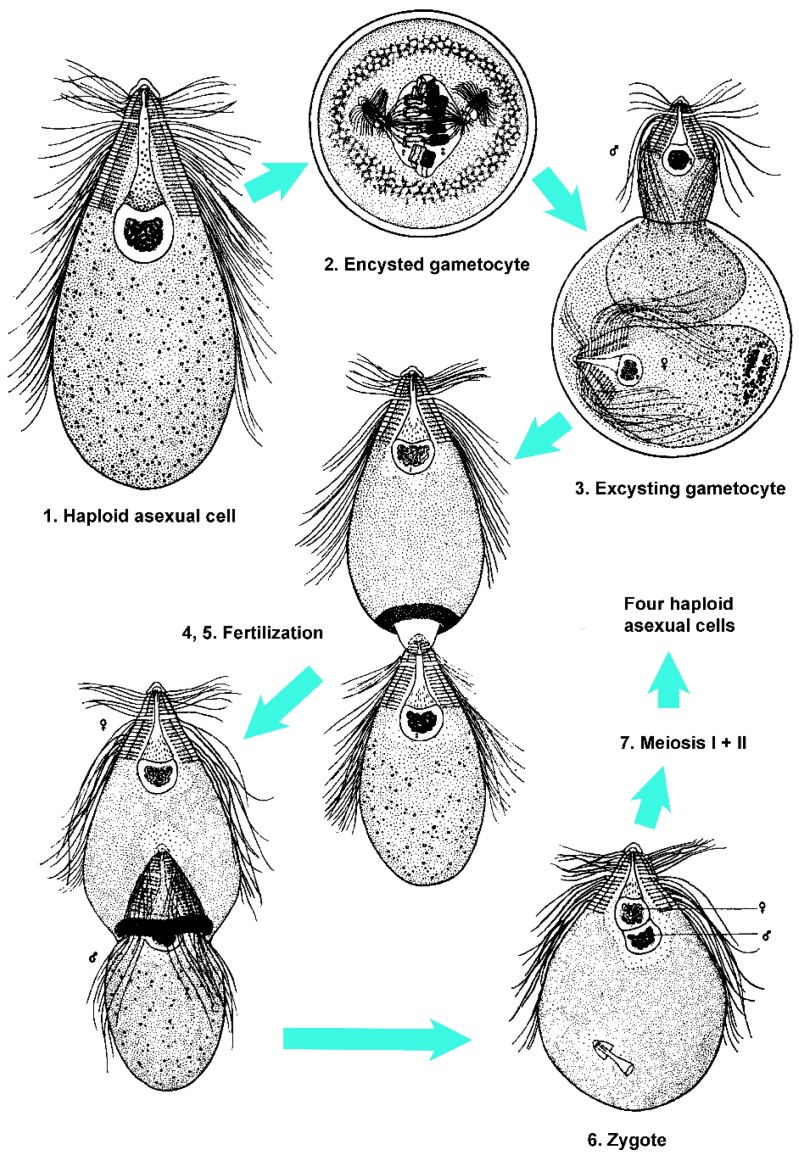
Sexual/encystment (SE) cycle in *Trichonympha*. After Figure A in Cleveland [167], with permission from John Wiley and Sons.

There is plenty of opportunity to address several pivotal aspects of this host-symbiont system. A good foundation lies in linking the regulation of developmental pathways in the host to the fate of the flagellates in its gut, and can be examined at a variety of levels. Cleveland’s (Figure 1) meticulous studies were based primarily on microscopic and film studies of the flagellates, and on transfaunation and hormone injection experiments. He cautioned that it is necessary to make in some cases several thousand slide preparations to find all stages of the SE cycle in a single genus of these flagellates, and made more than 20,000 slides during his studies of those in *Cryptocercus* [82,127]. To characterize the SE cycle of *Trichonympha* (Figure 5), he studied two complete sets of fixed and stained preparations at 15-min intervals from the beginning to the end of the sexual cycle (note: the cycle can last almost eight days—Figure 3). He additionally conducted extensive examinations of live *Trichonympha* using phase contrast, which he considered essential to rule out artifacts in the stained material [61].

The action of insect hormones and their precursors are considerably better known since Cleveland’s investigations, and molecular methods now allow a much deeper exploration of unseen underlying processes. Potentially productive directions for exploring the mechanistic basis of flagellate death at molt include characterizing the hormonal differences between young *Cryptocercus* and lower termite workers during their respective molting cycles; such data would help determine how the precise endocrine cascade to which the flagellates were adapted shifted when their host developed eusociality. Recent studies are beginning to lay the groundwork. Genes related to the synthesis, degradation, sequestration and reception of juvenile hormone have been detected in *Cryptocercus punctulatus* [158] and a common JH signaling pathway may regulate molting in this cockroach and in the termite *Zootermopsis nevadensis* [159]. A comparison of the endocrine underpinnings of the imaginal molt in this cockroach and lower termites would help illuminate responses of the gut flagellates during the final molting period.

Instead of relying on visual evaluation of whether a flagellate enters an SE cycle, molecular techniques now make it possible to detect both encystment and sexual processes. Every microbial cryptobiotic state is the direct result of the opening and closing of specific genes [129], and significant advances have been made in the molecular basis of encystation in pathogenic intestinal parasites. The pathway in the flagellate *Giardia lamblia*, for example, is well characterized, as cysts are a key virulence mechanism allowing it to survive in the environment and infect new hosts [102,160,161,162]. The encystment process is also well studied in ciliates [163,164,165,166].

Although Cleveland detected sexual cycles in flagellates of naturally molting termites, it took extraordinary effort on his part (Table 2). Molecular techniques now make the study of these events more tractable. The list of eukaryotic species that lack direct evidence for meiotic sex but appear sexual as suggested by the presence of meiosis-related genes is growing at a rapid rate [168]. Cryptic sex has been detected in dinoflagellates [169], *Giardia* [170], amoebae [171], and significantly, the parasite *Trichomonas vaginalis* [172], which is related to the parabasalids in *Cryptocercus* and lower termites. The genome of flagellates in *Cryptocercus* can be inventoried and used to develop a SE cycle ‘detection toolkit’ [173] to determine if these genes are conserved and expressed during molt in related flagellates of termites. This would allow characterization of the SE cycle during the early ‘conditioning’ period, when the process has been initiated, no visible changes are evident in the protists, but they are nonetheless vulnerable to hormonal shifts.

Because of the variation in SE cycles of the flagellates, efforts might best be directed to a single, well-studied flagellate genus such as *Trichonympha*, which predates the *Cryptocercus*-termite divergence [174]. There are seven species of *Trichonympha* in *Cryptocercus*, and the genus is found in at least 111 lower termite species [15,20,175]. It produces a well-formed cyst during the molting cycle of *Cryptocercus*, and all species in the cockroach go through precisely the same sexual cycle within the same time frame [15,61] (Figure 5). Moreover, Cleveland found traces of the SE cycle in two *Trichonympha* species from termites, including instances of *T. magna* surviving the imaginal molt in *Porotermes* (Table 2).

Symbiosis has entered a new era, with high throughput sequencing making it possible to analyze the capacities, functions and metabolism of each partner; analysis of the expression of mRNA or proteins further allow comparison of how symbiotic systems react when exposed to different environments [46]. These techniques will be crucial in uniting the functional and evolutionary biology of the *Cryptocercus*-termite lineage. In 1949, Cleveland [61] wrote that it would be a long time before an accurate evaluation could be made of the role of their hosts in initiating sexual cycles in their protozoa, in the alteration of the cycles once begun, and in protozoan evolution. Nearly 70 years later, it is time to revisit the problem.

## Figures and Tables

**Table 1:** Comparison of the host-flagellate relationship in a hypothetical cockroach ancestor, the wood-feeding cockroach *Cryptocercus,* and the lower termites. Characteristics of *Cryptocercus* are assumed to be the state immediately ancestral to lower termites. Key differences between *Cryptocercus* and lower termites are in red.

	Hypothetical Gregarious Ancestor	Subsocial *Cryptocercus*	Eusocial Lower Termites
Depends on flagellates to metabolize a cellulose-based diet	no	yes	yes
Parent to offspring vertical transmission of flagellates via trophallaxis	no	yes	yes ^a^
Flagellates die prior to molt of juvenile host	no	no	yes
Juveniles capable of solitary living	yes	yes ^b^	no
Post-molt transmission among family members via trophallaxis	no	no	yes
Transmission of flagellates via cysts in feces	yes	no ^c^	no
Transmission of flagellates via shed hindgut linings ^d^	no	no	no

^a^ During the subsocial stage of colony foundation. ^b^ After third instar. ^c^ Occasional horizontal transmission among siblings after initial vertical transmission of symbiosis is possible. ^d^ Either their own or that of a nest mate.

**Table 2:** Evidence for sexual/encystment (SE) cycles in the flagellates of termites. In each case except *Trichonympha magna* in *Porotermes adamsoni* alates, the cycles abort and the flagellates die prior to molt.

Flagellate	Termite Host	Reference	Notes
*Deltotrichonympha*	*Mastotermes darwiniensis*	[135]	Aspects of SE cycle similar to *Barbulanympha* and *Trichonympha*
*Koruga bonita*	*Mastotermes darwiniensis*	[136]	SE cycle similar to *Deltotrichonympha*
*Mixotricha paradoxa*	*Mastotermes darwiniensis*	[137]	Unique SE cycle
*Trichonympha magna*	*Porotermes adamsoni*	[138]	Studied in 3000 termites; in juvenile molt, all die; imaginal (alate) molt, a few survive.
*Trichonympha turkestanica*	*Anacanthotermes ochraecus*	[138]	Two instances of fertilization noted
*Pseudotrichonympha sp.*	*Prorhinotermes simplex*	[139]	SE cycle similar to *Trichonympha magna*
*Pseudotrichonympha hertwigi*	*Coptotermes acinaciformis*	[139]	Brief mention
*Pseudotrichonympha parvipapillosa*	*Schedorhinotermes intermedius*	[139]	Brief mention
*Pseudotrichonympha*-like	*Archotermopsis*	[133]	Brief mention
